# Sex, Endothelial Cell Functions, and Peripheral Artery Disease

**DOI:** 10.3390/ijms242417439

**Published:** 2023-12-13

**Authors:** Siân P. Cartland, Christopher P. Stanley, Christina Bursill, Freda Passam, Gemma A. Figtree, Sanjay Patel, Jacky Loa, Jonathan Golledge, David A. Robinson, Sarah J. Aitken, Mary M. Kavurma

**Affiliations:** 1 Heart Research Institute, The University of Sydney, Sydney, NSW 2042, Australia; sian.cartland@hri.org.au (S.P.C.); christopher.stanley@hri.org.au (C.P.S.); sanjay.patel@hri.org.au (S.P.); 2 South Australian Health and Medical Research Institute, Adelaide, SA 5000, Australia; christina.bursill@sahmri.com; 3 Adelaide Medical School, University of Adelaide, Adelaide, SA 5005, Australia; 4 Faculty of Health and Medicine, The University of Sydney, Sydney, NSW 2050, Australia; freda.passam@sydney.edu.au (F.P.); gemma.figtree@sydney.edu.au (G.A.F.); sarah.aitken@sydney.edu.au (S.J.A.); 5 Kolling Institute of Medical Research, Sydney, NSW 2064, Australia; 6 Royal Prince Alfred Hospital, Sydney, NSW 2050, Australiadavid_robinson@me.com (D.A.R.); 7 Queensland Research Centre for Peripheral Vascular Disease, College of Medicine and Dentistry, James Cook University, Townsville, QLD 4811, Australia; Jonathan.golledge@jcu.edu.au; 8 Department of Vascular and Endovascular Surgery, The Townsville University Hospital, Townsville, QLD 4814, Australia; 9 Concord Institute of Academic Surgery, Concord Repatriation General Hospital, Sydney, NSW 2139, Australia

**Keywords:** peripheral artery disease, endothelial cell dysfunction, sex differences

## Abstract

Peripheral artery disease (PAD) is caused by blocked arteries due to atherosclerosis and/or thrombosis which reduce blood flow to the lower limbs. It results in major morbidity, including ischemic limb, claudication, and amputation, with patients also suffering a heightened risk of heart attack, stroke, and death. Recent studies suggest women have a higher prevalence of PAD than men, and with worse outcomes after intervention. In addition to a potential unconscious bias faced by women with PAD in the health system, with underdiagnosis, and lower rates of guideline-based therapy, fundamental biological differences between men and women may be important. In this review, we highlight sexual dimorphisms in endothelial cell functions and how they may impact PAD pathophysiology in women. Understanding sex-specific mechanisms in PAD is essential for the development of new therapies and personalized care for patients with PAD.

## 1. Introduction

Peripheral artery disease (PAD) is a disease with high human and social impact, significantly reducing the quality of life. In this condition, an impairment of the blood supply due to atherosclerosis results in ischemia, most commonly to the lower limbs. More than 230 million people are affected by PAD globally [1], with the prevalence expected to increase because of a rise in diabetes mellitus [2]. In severe cases, patients, particularly those with diabetes, develop gangrene, which necessitates surgical amputation of the limbs. In the United States alone, 185,000 limbs are amputated every year, and it is estimated that by 2050, >3.5 million U.S. citizens will live without a limb [3]. Although revascularization surgery can improve perfusion, the current interventions may be insufficient because of extensive disease. Furthermore, the underlying atherosclerotic disease remains, and patients with PAD frequently undergo multiple vascular surgical procedures, each of which increases the risk of heart attack and stroke [4]. Indeed, patients who have undergone below- or above-knee amputations are more likely to die within 5 years; a mortality rate greater than those of breast, colon, and prostate cancer [5]. PAD, therefore, causes major trauma and disability, costing the U.S. economy USD 84–380 billion/year [6].

Remarkably, PAD does not affect society equally. Although reports are conflicting [7,8,9], the largest systematic review recently conducted highlights our under-recognition of the disease in women, with PAD more prevalent in women >25 years of age in high-income countries. Further, a higher proportion of women with PAD remain asymptomatic [10,11], which is associated with delayed presentation [12] and worse clinical outcomes post intervention [13,14]. We recently highlighted sex-specific disparities in PAD through a social constructivist perspective [15]; however, the biological differences for the clinical observations are unclear, revealing a need to understand sex-specific PAD pathophysiology. The endothelium could be key to explaining some of these differences.

The endothelium is a monolayer of endothelial cells (ECs) which lines the entire vascular tree, playing a critical role in maintaining cardiovascular homeostasis including regulating permeability, blood flow, vessel tone, inflammation, platelet function, and angiogenesis. Because EC dysfunction occurs early and progresses over the course of atherosclerosis development in PAD [16], in this review we discuss the sexual dimorphisms of EC function(s) that may impact PAD pathophysiology, particularly in women. This knowledge could have significant implications for sex-dependent therapeutic and diagnostic approaches for this disease.

## 2. Sexual Dimorphisms in EC Functions(s) in PAD

**Figure 1:**
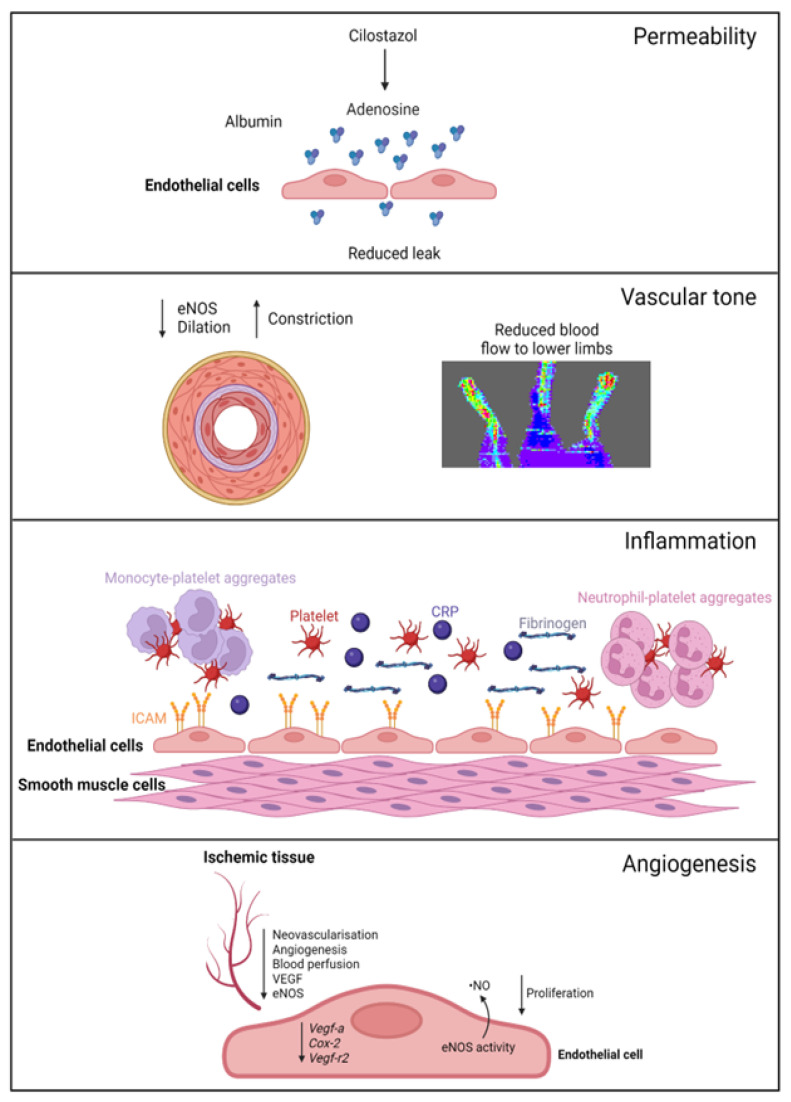
Female-specific findings from experimental models. *Permeability*: cilostazol increases adenosine levels, reducing leak in female, but not male, vessels. *Vascular tone*: female ischemic limbs express less eNOS (endothelial nitric oxide synthase) and have reduced arterial relaxation and increased arterial constriction compared to male ischemic limbs. *Inflammation*: female ECs have increased ICAM (intracellular adhesion molecule 1) expression. Females with PAD have increased levels of platelets, leukocyte–platelet aggregates, CRP (C-reactive protein), and fibrinogen in their circulation. *Angiogenesis*: female ECs have reduced proliferation and reduced expression of genes regulating angiogenesis, with angiogenic sprouting and migration in female cells specifically reliant on increased eNOS activity and ^·^NO (nitric oxide) release. Ischemic limbs in female mice have reduced expression of eNOS and VEGF (vascular endothelial growth factor) in tissues, with reduced angiogenesis and neovascularization. Created with BioRender.com (accessed on 9 December 2023).

**Figure 2:**
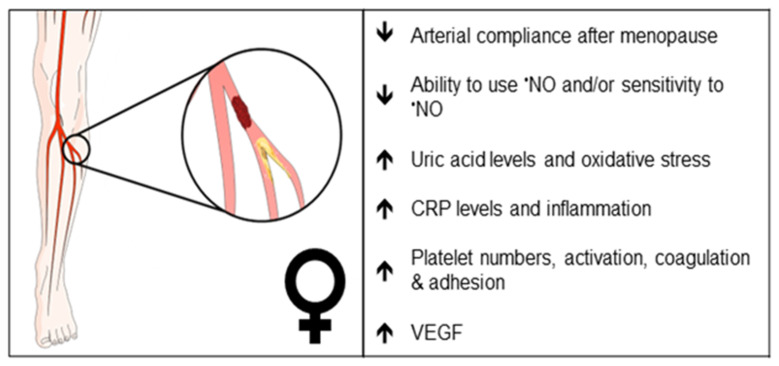
Clinical observations that may reflect differences in endothelial function in female PAD patients. Arrows indicate increase or decrease. •NO, nitric oxide; CRP, C-reactive protein; VEGF, vascular endothelial growth factor.

A summary of EC function(s) that could impact female PAD pathophysiology described by experimental models is shown in Figure 1. Clinical observations that may reflect differences in EC function(s) in female PAD patients are described in Figure 2. Both experimental and clinical findings are detailed in the text below.

### 2.1. Pathogenesis

PAD is traditionally thought to be caused by atherosclerosis; however, a recent study reported that 66% of large peripheral arteries examined from patients with chronic limb-threatening ischemia (CLTI), the severe form of PAD, were blocked by thrombus, in the absence of significant atherosclerosis [17,18]. Thrombi within smaller vessels were also identified [17,18]. Thrombosis may play a role in determining PAD disparities, since women have a higher platelet count than males, and female platelets have a higher reactivity than males [19]. Women also have more lesions in smaller vessels and multilevel disease [13]. Peripheral arteries from patients present with greater medial calcification and calcified nodules [17,18]; these may promote rupture by disrupting the fibrous cap. Interestingly, calcified nodules were present in 8% of coronary plaques from women ≥50 years of age vs. 3% of men in the same age bracket [20], and it is tempting to speculate that sex differences in calcification in peripheral arteries may exist; however, the authors found no differences in relation to sex. Additional studies are needed to understand the impact of sex and its role in atherosclerosis and/or thrombosis and in microvascular and multi-level disease.

### 2.2. Biomechanical Considerations

Blood vessels from women tend to be smaller in diameter than those of men, including blood vessels of the leg such as the common femoral artery [1] and vein [2], even when corrected for body weight. EC size may impact the vessel diameter; however, studies are conflicting. For example, male lung ECs isolated from mice were smaller than female ECs, whereas male ECs isolated from rat aorta were larger than the corresponding ECs from female rats [3,4]. Importantly, the smaller vessel size in females may contribute to arterial shear stress, which can influence vascular remodeling, restenosis after revascularization, and arterial compliance [5,6,7]. The smaller vessel size in women may also contribute to difficulties in revascularization [8], increased complications, and mortality. This is particularly evident following percutaneous coronary intervention and bypass grafting in women with coronary artery disease (CAD) [9,10]. The same may hold true for PAD. Women with CLTI have an increased rate of major adverse cardiovascular events and increased mortality after surgical revascularization or amputation [11,12]. Differences in sex hormones may also affect arterial compliance, with post-menopausal females having increased arterial stiffness compared to males [13], a finding that is further exaggerated in conditions such as metabolic syndrome [14]. Arterial stiffness contributes to the increased prevalence of hypertension in women [15], and hypertension is a risk factor for PAD [16]. These differences in the biomechanical properties of arteries may affect the sex-dependent outcomes of PAD treatment. Whether altered EC functions contribute to differential response and recovery post revascularization or surgery in women with PAD, particularly post-menopause women, remains unclear.

### 2.3. Vascular Tone

Sex differences in endothelial-dependent vasodilation are apparent [21], with young healthy female arteries showing increased arterial dilator responses to flow-mediated dilation (FMD; a surrogate of endothelial function) and chemical stimuli through enhanced nitric oxide- (·NO), cyclooxygenase- (COX), and/or hyperpolarization-dependent pathways [22]. In part, this is attributed to increased levels of estrogen during the menstrual cycle. Interestingly, female vessels show greater dependence on ·NO-mediated arterial relaxation, but with aging and loss of estrogen (i.e., menopause), these responses are lost (extensively reviewed in [23,24]). In Table 1, we summarize sex-dependent mechanisms mediating vessel tone in multiple vascular beds isolated from wildtype C57Bl6 mice. In preclinical PAD, in the hindlimb ischemia model, ischemic female limbs had reduced endothelial nitric oxide synthase (eNOS) protein expression, associating with decreased arterial relaxation to acetylcholine, greater resistance to flow, and increased arterial constriction when compared to male limbs [25]. Ischemic female limbs also had reduced blood perfusion to the lower limbs [25]. In humans, systemic ·NO synthesis rates were significantly lower in PAD patients (stage II–IV) compared to control subjects, but not significantly different between sexes [26]. Interestingly, nitrate and cyclic guanosine monophosphate (cGMP) excretion rates were higher in female than in male subjects (with the trend reaching statistical significance), implying altered ·NO signaling in women with PAD, a finding not further elaborated upon by the authors [26]. These studies suggest that females may produce sufficient ·NO but are unable to utilize it for vasodilatory purposes or that the sensitivity of female vessels to ·NO is reduced.

Changes in oxidative stress may also contribute to vessel tone. Uric acid is an end-product of purine metabolism and the most plentiful antioxidant in plasma. High levels of uric acid are associated with cardiovascular diseases including PAD [27,28] and with endothelial dysfunction, in part, by reducing ·NO bioavailability [28,29]. Taher and colleagues performed a retrospective cross-sectional analysis on peripheral microvascular dysfunction (reactive hyperemia peripheral arterial tonometry via Endo-PAT) and serum uric acid levels ≥5 mg/dL in cardiovascular disease patients. The authors identified a significant positive association between the two, and specifically, the association was only observed in women [30]. Whether this is a possible mechanism for impaired ·NO bioavailability in women with PAD remains to be demonstrated.

**Table 1:** Sex differences in the mechanisms of relaxation in C57Bl6 mice.

Stimulus	Age	Gender	Artery	Mechanism of Relaxation	Ref.
Ach	8 Weeks	Male	Mesenteric (150 µm)	~60% ^·^NO-/COX-dependent ~20% IK_ca_-/SK_ca_-dependant ~20% Unknown	[31]
	8 Weeks	Female	Mesenteric (150 µm)	~40% ^·^NO-/COX-dependent ~40% IK_ca_-/SK_ca_-dependant ~20% Undetermined	[31]
	6–8 Weeks	Male	Superior mesenteric	~70% ^·^NO-/COX-dependent ~30% Undetermined	[32]
	Any	Female	* Superior mesenteric	No data	
	6–8 Weeks	Male	Thoracic aorta	100% ^·^NO-dependent	[32]
	8 Weeks	Female	Thoracic aorta	100% ^·^NO-dependent	[33]
	Not specified	Male	Carotid	~50% ^·^NO-/COX-dependent ~50% Undetermined	[34]
	19–23 weeks	Female	Carotid	100% ^·^NO-dependent	[35]
	Not specified	Male	Femoral	~60% ^·^NO-/COX-dependent ~40% Undetermined	[34]
	19–23 weeks	Female	Femoral	~75% ^·^NO ~25% BK_ca_/IK_ca_/SK_ca_	[35]
Flow	10–14 weeks	Male	Cerebral	~50% ^·^NO-dependent ~50% H_2_O_2_-dependent	[36]
	Any	Female	* Cerebral	No Data	
	5–6 months	Male	Mesenteric (200 µm)	~50% ^·^NO-dependent ~50% Undetermined	[37]
	Any	Female	* Mesenteric	No data	
	26 months	Male	Femoral	~100% ^·^NO-dependent	[38]
	Any	Male	* Femoral	No data	

Ach, acetylcholine; ·NO, nitric oxide; COX, cyclooxygenase; IK_ca_, intermediate-conductance calcium-activated potassium channel; SK_ca_, small-conductance calcium-activated potassium channel; BK_ca_, big-conductance calcium-activated potassium channel. * No data found in the literature assessing relaxation in these arteries.

### 2.4. Barrier Function and Permeability

The evidence that barrier function differs in males and females with PAD is weak. However, there are two strands of evidence that point to altered features depending on sex. A recent study examined male and female CAD patients; both sexes had significantly impaired perfusion in their microvasculature, measured as a percentage of microvascular vessels occupied by red blood cells [39]. Females had deeper penetration of red blood cells into the sublingual glycocalyx, indicating greater impairment in barrier function when compared to males [39]. Whether a similar disfunction is observed in women with PAD is yet to be established.

Phosphodiesterases (PDEs) play an important role in barrier function, as they inactivate the cyclic nucleotide messengers cyclic adenosine monophosphate (cAMP) and cGMP. ECs are known to express five PDEs, namely, PDE1, PDE2, PDE3, PDE4, and PDE5. Cilostazol is a PDE3 inhibitor, and an anti-platelet medication used to relieve PAD patients with symptoms of intermittent claudication. Cilostazol treatment was shown to improve walking distance [40], in part, via its ability to act as a vasodilator. It can also increase adenosine concentrations in patients with acute coronary syndromes [41] and reduce permeability [42], and, particularly relevant to this review, it reduced permeability in female, but not in male, microvascular ECs [43]. Interestingly, female microvascular ECs express more *Pde3b* mRNA than male ECs [43], implying that cilostazol may have sex-dependent actions.

### 2.5. Leukocyte Trafficking and Inflammation

How inflammatory cells and molecules relate to sex differences in the presence of PAD is unclear. Sex differences in immune responses have been described [44] and are influenced by the environment, genetic mediators, and hormones. With regard to the latter, the presence of estrogens, particularly, 17β-estradiol in premenopausal women, is thought to be protective [45]. Indeed, premenopausal women have a reduced prevalence of CAD, hypertension, myocardial infarction, and stroke compared to men; however, the prevalence of these conditions in women after menopause surpasses that of men [46]. This protection is in part due to the anti-inflammatory and antioxidant properties of female sex hormones (i.e., estrogens), which are lost after menopause [23]. However, the benefit of estrogen therapy in post-menopausal women is conflicting and reflects the fact that the contribution of sex hormones to inflammation, oxidation, and atherosclerosis is complex and influenced by the effects of the sex chromosomes [47] and age- and sex-dependent differences in specific organs and tissues [23]. What is known is that women with PAD have ~1.4 times higher levels of CRP than men, associating with greater PAD prevalence [48]. Women also have higher levels of CRP and fibrinogen and are more likely to present with CLTI following autogenous vein lower extremity bypass, which is associated with graft failure, with the authors proposing an impaired healing response in women to account for this [49].

Sex differences also appear to impact EC inflammatory marker expression. For example, female skeletal ECs show greater expression of intercellular adhesion molecule-1 (ICAM-1) than male skeletal ECs under basal conditions, whereas vascular cell adhesion molecule (VCAM-1) is expressed in male ECs to a greater extent [50]. This is somewhat supported by other studies; women with PAD of African American descent had elevated levels of ICAM-1, whereas Caucasian women had increased levels of MMP-9 and VCAM-1 when compared to men with PAD from each race [51]. Furthermore, ECs exposed to sera from African American women showed significantly increased intracellular oxidative stress compared to ECs exposed to sera from male African Americans [51]. Importantly, the same authors found that women may have a weakness in their ability to increase capillary blood volume following exercise treatment, since their time to reach minimal calf muscle oxygen saturation levels—an important measure of microcirculatory function—was significantly shorter than men [52]. These findings suggest that anti-inflammatories and medications that improve EC function may be beneficial in women with PAD.

In inflammatory and thrombotic conditions, platelet–leukocyte aggregates can form, enhancing platelet activation. A small study identified that the levels of stimulated platelet–neutrophil aggregates were significantly increased in diabetic women with and without cardiovascular disease in comparison to men with the same condition [53]. Increased suppressor of cytokine signaling 3 (SOCS3) expression was also associated with increased circulating monocyte–platelet aggregates in women that had a myocardial infarction [54]. These observations support the notion that increased platelet number and activity, as well as their increased interaction with leukocytes, is linked to increased adhesivity to the endothelium and to increased aggregability. Coagulation factors such as tissue factor can also promote inflammatory responses by activating protease-activated receptors [55]; however, sex-dependent effects are unclear. The crosstalk between inflammation and coagulation factors contributing to PAD pathogenesis, recently reviewed elsewhere [56], highlights the complexity of this system. More studies are needed to fully appreciate the impact of sex-dependent EC thrombo-inflammatory processes in PAD.

### 2.6. Platelets and Coagulation

The endothelium provides a surface for the assembly of platelets and coagulation factors and for the development of thrombosis, which is a common complication of PAD. Females have an overall higher platelet count compared to males, and as mentioned earlier, they have a higher baseline reactivity [19]. Female platelets show consistently higher aggregation in response to agonists, such as arachidonic acid, collagen, and adenosine diphosphate (ADP) [57]. In contrast, platelets from healthy female subjects show less baseline platelet adhesion to the endothelium compared to male platelets [58]. This difference in platelet reactivity may be due to sex hormones. Both female and male platelets express receptors for 17β-estradiol as well as androgen and progesterone receptors; however, their effect on platelet function is somewhat controversial [59,60]. Platelet receptors can also regulate platelet reactivity in a sex-dependent manner. Platelet adhesion and thrombus formation is dependent on platelet glycoprotein receptors including glycoprotein IIb/IIIa (fibrinogen receptor), Iba (von Willebrand factor receptor), and VI and a2b1 (collagen receptors). Platelet activation and aggregation is mediated through the G-protein receptors PAR1 (protease activated receptor 1) and PAR4 (thrombin receptors), P2Y12 receptors (ADP receptors), and the thromboxane A2 receptor, amongst others [61]. While there is no difference in the expression of glycoprotein IIb/IIIa between sexes, women show higher receptor reactivity [59]. Moreover, healthy women and female mice have higher reactivity to PAR1 and PAR4 agonists [62], suggesting sex-dependent expression or activation of these receptors.

Circulating coagulation factors are influenced by hormonal differences in females vs. males. The most outstanding example of this is pregnancy, where multiple coagulation factors are upregulated, presumably as an evolutionary mechanism to prevent maternal death from post-partum hemorrhage. Women have higher average levels of von Willebrand factor and factor VIII [63], with further increases in von Willebrand factor and fibrinogen concentrations developing during pregnancy. In contrast, pre-menopausal women have lower levels of the antithrombotic proteins, protein S and protein C, and conversely, lower levels of factor X, an enzyme of the coagulation cascade [64]. Not surprisingly, elevated von Willebrand factor and fibrinogen levels are independently associated with the risk of development of PAD [65]. Fibrinolytic activity is also a feature of PAD with poor outcomes. Women with CAD have high levels of PAI-1 [66], which may indicate impaired fibrinolysis. The increase in PAI-1 was further exaggerated in females with type-2 diabetes [67]. These studies indicate that females with cardiovascular disease, including PAD, have altered platelet function, coagulation, and fibrinolysis, which may contribute to worse outcomes.

In terms of current therapies, females display higher baseline platelet reactivity, which contradicts the anti-aggregatory effect of aspirin [19]. In a study of low-dose aspirin therapy in unaffected individuals from families with premature coronary disease, women had consistently more reactive platelets compared with men, to multiple agonists at baseline, which persisted after aspirin therapy [19]. It must be noted that despite sex differences being statistically significant, the magnitude of the differences was small. Another study with low-dose aspirin, showed that daily aspirin exposure resulted in a paradoxical attenuation of platelet inhibition in response to epinephrine and ADP over time in women but not in men [57]. The second most common anti-platelet agent, clopidogrel, also showed a differential effect in females versus males. Women on clopidogrel, evaluated prior to cardiac surgery, had higher reactivity to ADP compared with men [68]. Inadequate antiplatelet responses to fixed doses in women may support the rationale of sex-tailored agent selection or dosage. In terms of the side effects of anti-platelet treatments, there is some evidence for sex differences; a multivariate analysis confirmed that newer antiplatelet agents are an independent risk factor for bleeding only in women [69]. How these findings relate to EC sex-dependent differences in patients with PAD require further elucidation.

### 2.7. Angiogenesis

Angiogenesis is a critical physiological process in which new blood vessels grow from a pre-existing vessel bed. These neovessels are the cornerstones of nutrient diffusion, essential in tissue development and wound repair. Increasing evidence suggests sex differences in angiogenic responses in ECs. For example, female microvascular ECs isolated from skeletal muscle appeared to grow more slowly than male cells [50]. In a rat cornea model, neovascularization was significantly reduced in females compared to males, across a range of rat strains [70], and this was an androgen-independent response [70]. Rather, the expression of cyclooxygenase-2 (Cox-2), vascular endothelial growth factor-A (VEGF-A), and vascular endothelial growth factor-receptor 2 (VEGF-R2) was higher in male than in female ECs, suggesting enhanced angiogenic priming in male ECs. In porcine valvular ECs, male cells exhibited higher proliferation rates vs. female cells, which was associated with increased secretion of pro-angiogenic VEGF-A, platelet derived growth factor (PDGF), and endothelin-1 from neighboring male interstitial cells, whereas female interstitial cells secreted greater levels of anti-angiogenic factors [71]. In contrast, other studies either reported no differences in angiogenic processes or showed increased angiogenic capacity in female ECs compared to male ECs in vitro, associated with increased platelet endothelial cell adhesion molecule-1 (PECAM-1) or eNOS expression [72,73,74]. Interestingly, female (but not male) EC angiogenic sprouting and migration were found to be reliant on increased eNOS activity and ·NO release [75,76].

Angiogenesis is considered a key protective mechanism against symptomatic PAD—including against the susceptibility to claudication, ischemic ulcers, and limb amputation. Sex-dependent changes in angiogenesis have been described in mouse models of PAD, where reduced angiogenesis and capillary density were associated with impaired blood perfusion in female vs. male C57Bl6 ischemic hindlimbs [25]. The authors found that the basal VEGF and eNOS protein expression was greater in female limb tissues, whereas 7 days post-ischemia, male tissues displayed greater VEGF and eNOS expression [25]. Sex hormones also play a role, since estrogen-related receptor-α expression is induced in the ischemic hindlimb of male mice, which is associated with increased vascularization and non-leaky blood vessel formation [77]. Further, oophorectomized mice showed reduced neovascularization and eNOS protein expression after hindlimb ischemia when compared to female control mice [78]. A clinical study assessing 234 patients (145 males, 89 females) with PAD and 50 healthy controls reported higher levels of plasma VEGF in female vs. male PAD patients [79]. Given that VEGF is increased in ischemia and high levels of VEGF predict PAD progression and severity [80], disease may indeed be far worse in females.

## 3. Lessons from OMIC Studies

Multi-omic approaches could be a powerful tool in deciphering mechanisms of pathogenesis and in identifying potential therapeutic targets for sex-specific medicine in PAD patients. Interestingly, 14–25% of the EC transcriptome is reported to be sex-specific [81]. Recently, single-cell RNA sequencing data from the Tabula Muris Consortium interrogated EC transcriptomes from 12 organs including the limb [82]. *Lars2*, encoding the enzyme leucyl-tRNA synthetase 2, important in mitochondrial protein synthesis, was identified by two independent studies to be expressed in male ECs, with low expression in female cells [82,83]. These findings highlight possible sex differences in the function of EC mitochondria. Indeed, male microvascular ECs have ~1.7 times greater basal mitochondrial respiration and higher adenosine triphosphate (ATP) production when compared to female cells, suggesting that male microvascular ECs may have more functional mitochondria [84]. When challenged with hypoxia (2% O_2_) or in response to antimycin A (a specific inhibitor of complex III in the mitochondrial electron transport chain), female ECs died more rapidly than the male cells [84]. However, these data were not reported for macrovascular human umbilical vein ECs isolated from dizygotic twins, where no differences in cellular energy production (glycolysis versus mitochondrial respiration), intracellular ATP, or metabolite levels were identified [85], revealing not only sex-dependent, but also microvascular-vs.-macrovascular EC differences. These observations highlight that EC behavior is governed by their microenvironment and the organs/tissues that they reside in. Nevertheless, these data provide mechanistic insight into EC function and that female microvascular ECs respond differently to mitochondrial stress. Mitochondrial dysfunction is apparent in the skeletal muscle of patients presenting with intermittent claudication [86] and in mouse models of PAD [87]. In-depth transcriptomic and proteomic analyses were recently conducted in muscle biopsies from people with and without PAD, identifying an accumulation of mitochondrial proteins in PAD tissue, reduced levels of glycolytic enzymes, and increased levels of proteins necessary for stress-induced protein translation [88]. Sex differences were not described here. Whether EC mitochondrial function plays a role in sex differences in PAD patients requires further study.

## 4. Risk Factors and microRNAs

Smoking and diabetes negatively impact the endothelium [89,90] and are considered two of the most significant risk factors in the pathogenesis of PAD. A systemic review in 2018 identified that half of all PAD cases were attributed to smoking [91]. Remarkably, passive smokers and ex-smokers also had increased risk of PAD [92,93]. Overall, men with PAD are more likely to smoke [94], but in a study of U.K. biobank participants, female smokers were found to have a greater risk of PAD when compared to male smokers [95]. A recent study investigating the effect of smoking on microRNA (miRNA) expression showed that *miR-27b* was downregulated in active smokers, associating with the presence and severity of PAD [96,97]. Interestingly, *miR-27b* was found to improve endothelial health by attenuating oxidative stress and inflammation [98]. miRNAs are small, single-stranded, non-coding RNA molecules that act as negative regulators of gene expression. That *miR-27b* is regulated by estrogen [99] suggests sexual dimorphism; however, its role in sex-dependent EC function(s) is PAD patients is yet to be elucidated.

Impaired glucose tolerance and diabetes increase the risk of developing PAD, increasing PAD severity and the need for amputation [100,101]. Even after controlling for major risk factors, diabetes was associated with a higher number of occlusive vascular deaths in women compared to men [102]. Several miRNAs were recently highlighted for their association with arterial occlusive disease, with *miR-134-5p* identified as a useful prognostic marker for patients with diabetes [103]. *miR-134-5p* is known to inhibit angiogenesis [104] and contribute to glucose-induced EC dysfunction [105]. Whether *miR-134-5p* impacts sex differences in EC functions in PAD patients remains to be determined.

## 5. Outstanding Questions and Future Perspectives

The global burden of PAD in women has been underappreciated. Our understanding of sex-dependent presentation, diagnosis, and treatment of PAD and our comprehension of disease pathophysiology are limited, and outstanding questions remain (Table 2). Further research efforts into PAD pathogenesis, particularly, sex-dependent differences, are essential, as this will dictate how we manage, support, and treat patients for optimum therapeutic benefit. Overall, the evidence points towards increased levels of immune molecules, immune cells, and platelets in women with PAD, and this, in combination with differences in EC function and oxidative stress, may contribute to the higher female prevalence and altered pathogenesis of the disease. Women, overall, have higher resistance to antiplatelet agents (aspirin and clopidogrel). They also have higher values of coagulation parameters. There is a pressing clinical need to investigate if the recently introduced combinations of anti-Xa anticoagulants and antiplatelet agents [106] are more effective in women compared to men and to establish their optimal doses. Based on the heightened response of female platelets and inflammatory cells to immune triggers, it would be important to investigate if female patients with PAD would benefit from the combination of anti-inflammatory treatments with traditional treatments. Sex differences in EC function(s) in PAD patients exist, revealing an area with unexplored diagnostic and therapeutic potential.

## Figures and Tables

**Table 2:** Outstanding questions on sex differences in PAD pathophysiology.

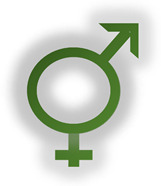	Is disease pathogenesis different between men and women? What are the genes, proteins, and pathways that mediate sex-dependent endothelial actions in homeostasis, and how are these altered in PAD? Can we target the endothelium for sex-specific therapeutic benefit? What is the contribution of sex hormones and sex chromosomes in PAD? Can we establish new physiological models of PAD to examine pathogenesis? What is the impact of diabetes-related PAD on sex-dependent EC functions?

## Data Availability

Data sharing not applicable.
